# Comparison of attitudes, awareness, and perceptions regarding oral healthcare between dental and nursing students before and after oral healthcare education

**DOI:** 10.1186/s12903-021-01554-8

**Published:** 2021-04-12

**Authors:** Satoru Haresaku, Yojiro Umezaki, Rui Egashira, Toru Naito, Keiko Kubota, Hidechika Iino, Hisae Aoki, Fuyuko Nakashima

**Affiliations:** 1 Department of Nursing, Fukuoka Nursing College, 2-15-1 Tamura, Sawara-ku, Fukuoka, 814-0193 Japan; 2 Section of Geriatric Dentistry, Department of General Dentistry, Fukuoka Dental College, 2-15-1 Tamura, Sawara-ku, Fukuoka, 814-0193 Japan

**Keywords:** Dental education, Oral healthcare education, Multi-professional education, Collaborative oral healthcare, Dental students, Nursing students

## Abstract

**Background:**

Oral healthcare education for health professional students is important to promote collaborative oral healthcare practice among health professionals. The purpose of this follow-up, cross-sectional study was to investigate attitudes, awareness, and perceptions regarding oral healthcare among dental and nursing students and to compare them both between baseline and the completion of the education programme and between dental and nursing students to identify problems with oral healthcare programmes in dental education.

**Method:**

The subjects included 88 dental and 119 nursing students. The dental students participated in geriatric and preventive dentistry courses for oral healthcare education. The nursing students participated in independent oral healthcare courses comprising 45 h of training with case-based learning and were taught and instructed by multiple health professionals, including dentists. Questionnaires were distributed to the participants to compare attitudes, awareness, and perceptions regarding oral healthcare between baseline and the completion of the education programme and between dental and nursing students. A chi-square test, Wilcoxon signed-rank test, and Mann–Whitney U test were used to compare the data.

**Result and Conclusion:**

The data of 48 (28 male and 20 female) dental students and 103 (9 male and 94 female) nursing students who completed the questionnaires both at baseline and after the education programme were used for the comparisons. After the education programme, more than 90% of the students were interested in oral healthcare practice; hoped to practise oral healthcare post-qualification; and perceived oral healthcare to be effective for preventing dental caries, periodontal diseases, and aspiration pneumonia. These attitudes and perceptions were statistically significantly improved after the education. However, the level of awareness of oral healthcare and the level of perception of the importance of collaboration with healthcare workers in oral healthcare practice after education were lower in the dental students than in the nursing students. Multi-professional oral healthcare education with case-based learning has the potential to improve awareness of oral healthcare and perceptions of the importance of collaborative oral healthcare practice.

**Supplementary Information:**

The online version contains supplementary material available at 10.1186/s12903-021-01554-8.

## Background

Oral healthcare is important for preventing not only dental diseases but also general diseases such as aspiration pneumonia, ventilator-associated pneumonia (VAP), diabetes, and cardiovascular disease [1–4]. In addition, recent studies have reported that oral healthcare is important in the prevention of viral infections, including COVID-19 infection [5–7]. Therefore, collaborative oral healthcare among health professionals is also important in preventing these diseases in patients in hospitals, older adults in long-term care facilities, and community dwelling older adults [8–14].

To promote collaborative oral healthcare in these contexts, oral healthcare education is needed for both oral health professionals and other health professionals, such as nurses, physicians, certified speech-hearing therapists, and certified care workers [15].

However, several studies have reported that interest in and knowledge of oral healthcare are lower in health professional students, such as nursing and medical students, than in oral health professionals, which might be obstacles for collaboration with oral health professionals in oral healthcare practice [16–20]. Therefore, oral health professionals have tried to address these problems and have reported that multi-professional education is effective in improving attitudes and perceptions regarding oral healthcare among health professional students [21–26]. One study reported that a total of 45-h oral healthcare courses with case-based learning conducted by multiple professionals were effective in improving attitudes towards oral healthcare among nursing students and that almost all of the students hoped to practice collaborative oral healthcare post-qualification [26].

On the other hand, a previous study on oral healthcare education in dental schools reported that there were no theoretical or practical courses on oral healthcare for older adults and that although some university departments, such as geriatric dentistry and preventive dentistry departments, teach some aspects of oral healthcare, they tend to do so without coordination and without formal courses in oral healthcare for older adults [27]. In addition, there have been no studies investigating attitudes, perceptions, and awareness among dental students or comparing them between dental and other health professional students after oral healthcare education.

The purpose of this study was to compare the attitudes, awareness, and perceptions regarding oral healthcare between dental students in their first and fourth years and between dental and nursing students to explore problems related to oral healthcare education in dental education.

## Methods

### Design and sample

**Fig. 1:**
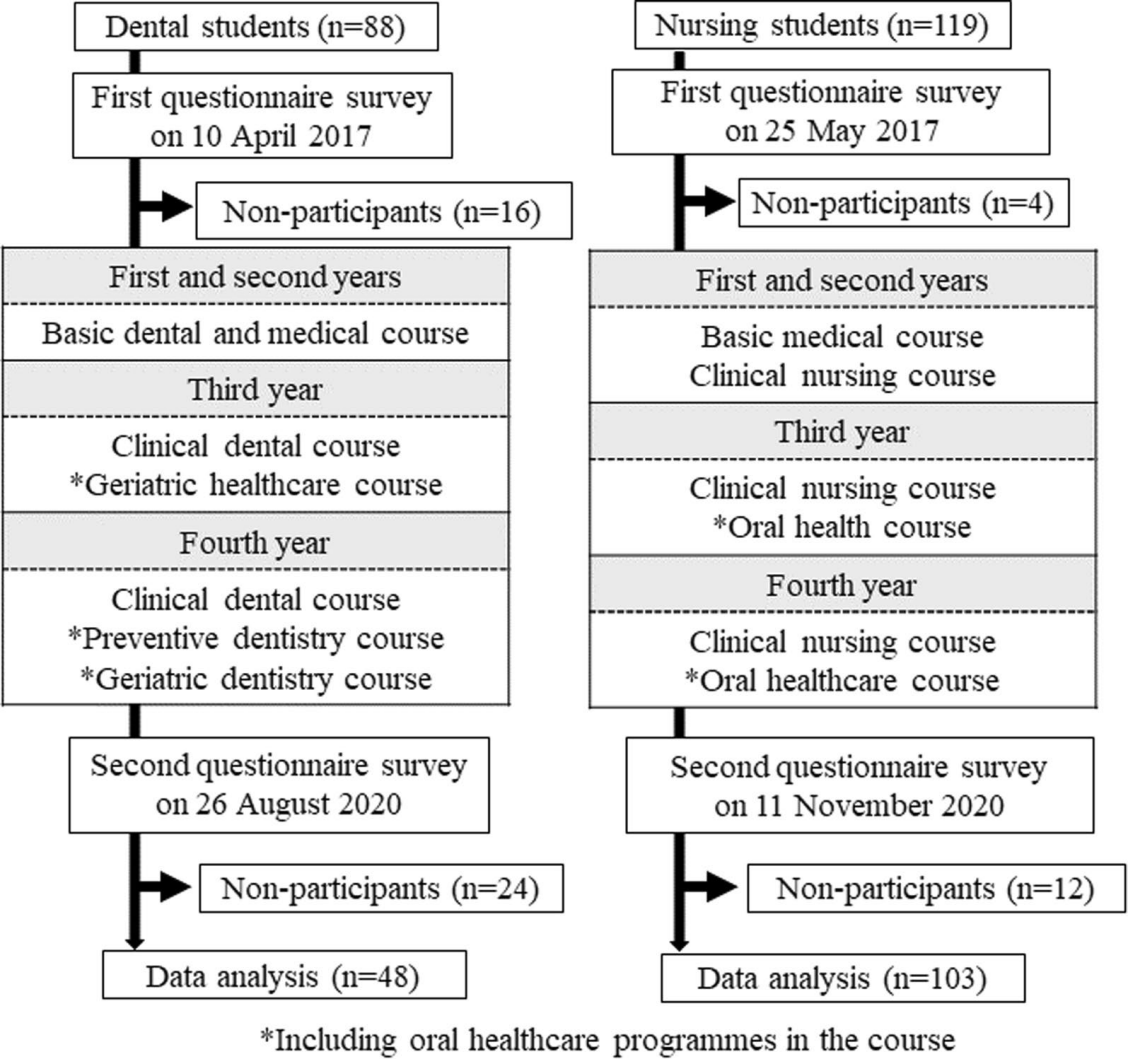
Study design in this study

This study was a 4-year follow-up, cross-sectional study (Fig. 1). The subjects included 88 dental students in a 6-year dental school and 119 nursing students in a 4-year nursing school as of April 2017. The dental and nursing schools belong to the same school cooperation in Fukuoka Prefecture, Japan. The nursing school enlists the cooperation of the dental school in oral healthcare education.

During the 4-year study period, the dental students participated in basic and clinical dental courses from the first to fourth years; however, they had not participated in the clinical practice training courses, which started in the fifth year (Fig. 1). For oral healthcare education, the students participated in a 22.5-h geriatric healthcare course (third-year course) from September to December 2019. Physicians and dentists lectured about healthcare provisions for acute, convalescent, and end-of-life patients in hospitals, disabled older adults in long-term care facilities, and community-dwelling older adults. Certified care workers instructed on the provision of care, including oral healthcare practice, for disabled older adults in training practice.

In addition, the dental students participated in a 45-h preventive dentistry course (fourth-year course) and a 22.5-h geriatric dentistry course (fourth-year course) from April to November 2020. In the courses, preventive dentists lectured and instructed how to practise toothbrushing and use interdental cleaning tools with patients,; dentists specializing in geriatric dentistry lectured about dental treatment and collaborative oral healthcare practice for older adult patients; a physician lectured about general diseases and the relationship between oral and general health; and a speech-hearing therapist lectured about training in speech, hearing, eating, and swallowing for patients.

The nursing students participated in a 22.5-h oral health course (third-year course) from September 2018 to January 2019 and a 22.5-h oral healthcare course (fourth-year course) in August 2020 (Fig. 1). Both courses were required courses and were collaboratively created by nurses and oral health professional staff in the dental and nursing schools. In the oral health course, a dentist specializing in geriatric dentistry lectured on oral anatomy, oral diseases, treatment of dental diseases, and the role of oral health professionals in oral healthcare teams. A speech-hearing therapist lectured on the role of speech-hearing therapists in healthcare teams and training in speech, hearing, eating, and swallowing for patients. A dental hygienist instructed the students on how to practise oral healthcare in practical training. A nurse specializing in dysphagia nursing lectured about the role of dysphagia nurses in healthcare teams and instructed how to support patients’ eating and swallowing in practical training.

The nursing students took the oral healthcare course via the internet from home due to the COVID-19 pandemic. They watched an online video created by a dentist specializing in preventive dentistry regarding the importance of collaborative oral healthcare and the use of oral healthcare tools. After watching the video, they self-practised oral healthcare with the tools that had been distributed to them via mail from the school and reported their thoughts about their use at home. They also watched online videos, which were created by dentists and nurses, regarding collaborative oral healthcare practice for patients with various general diseases in patients in paediatric, cancer, perioperative, convalescent, psychiatric, and palliative care hospital wards; older adults in long-term care facilities; and community dwelling older adults. After watching the videos, the students compiled case reports regarding oral healthcare practice.

### Structured questionnaires

The questionnaire was based on a previously developed questionnaire used to assess attitudes, awareness, and perceptions regarding oral healthcare among health professional students and healthcare workers [26, 28].

The questionnaire consisted of 4 parts: gender and age, attitudes towards oral healthcare practice, awareness of oral healthcare, and perceptions of the need to learn procedures in oral healthcare.

Regarding the participants’ attitudes towards oral healthcare, they were asked (a) if they were interested in oral healthcare practice, (b) if they wanted to practise oral healthcare post-qualification, and (c) what proportion of their duties would be dedicated to practising oral healthcare post-qualification. A five-point Likert response scale was used for (a) and (b), with the choices including “Very much”, “Somewhat”, “A little”, “Not very much”, and “Not at all”. A four-point Likert response scale was used for (c), with the choices “< 25%”, “25–49%”, “50–74%”, and “≥ 75%”. To evaluate the participants’ levels of interest for (a) and willingness for (b), the response choices were scored as “4”, “3”, “2”, “1”, and “0”, respectively. A score of 4 indicated the highest level of interest or willingness.

Awareness of oral healthcare was assessed in relation to four topics: the kind of knowledge needed to practise oral healthcare (5 items), to whom oral healthcare should be provided (6 items), where oral healthcare should be provided (10 items), and what aspects are affected by oral healthcare (8 items). These items were multiple-choice questions and contained “Other” as a response option. If participants chose “Other”, they were asked to specify their responses in writing. To determine the level of awareness of oral healthcare, the response options they chose were summed. The score ranged from 0 to 29 points.

Regarding perceptions of oral healthcare practice, the participants were asked (a) what kind of procedures or instructions in oral healthcare they needed to learn (14 items each for theory and practice) and (b) how healthcare workers are important for practising collaborative oral healthcare. Question (b) was asked only in the second survey, and a four-point Likert response scale was used, with the response options including “Very much”, “Somewhat”, “Not very much”, and “Not at all”. To evaluate the level of perception for (a), the item scores were summed. The scores for theory and practice each ranged from 0 to 14 points. In addition, the choices for (b) were scored as “3”, “2”, “1”, and “0” to evaluate the level of perception of the importance of collaboration with healthcare workers in oral healthcare practice. The score ranged from 0 to 3 points for each type of healthcare worker.

The participants were instructed not to select an option if they did not understand its meaning for all questions.

### Data procedure

The first and second questionnaire surveys were conducted on 10 April 2017 and 26 August 2020 in the nursing school and on 25 May 2017 and 11 November 2020 in the dental school, respectively (Fig. 1). After a lecture finished, the questionnaires were distributed to all the students who had attended the lecture. However, since the students in the nursing school were receiving lectures online due to the COVID-19 pandemic during the second survey period, they were asked to complete the second questionnaire within 2 weeks via the internet. The data for the nursing students were partially from a previous study that investigated the effectiveness of oral healthcare courses in improving nursing students’ attitudes and perceptions regarding oral healthcare [26].

All procedures performed in studies involving human participants were approved by the Ethics Committee of Fukuoka Gakuen, Fukuoka, Japan (approval No. 320) and were in accordance with the Ethical Guidelines for Clinical Research (the Ministry of Health, Labour and Welfare, Tokyo, Japan, No. 415 of 2008) and the 1964 Declaration of Helsinki and its later amendments or comparable ethical standards.

### Statistical analyses

A chi-squared test was used to compare the differences in attitudes, awareness, and perceptions regarding oral healthcare between baseline (when students were in their first year) and the completion of the oral healthcare education (when students were in their fourth year) in each student group and between the dental and nursing students. A Wilcoxon signed-rank test was used to compare the levels of attitudes, awareness, and perceptions between students in their first and fourth years, and a Mann–Whitney U test was used to compare them between dental and nursing students.

Data were analysed with 5% significance. The statistical analyses were performed using the IBM SPSS Statistics software program (Version 21.0; IBM Corporation, Armonk, NY, USA).

## Results

A total of 48 (54.5%) dental students and 103 (86.6%) nursing students completed questionnaires in both their first and fourth years. The majority of the dental students were male (58.3%), and the majority of the nursing students were female (91.2%). The mean age at baseline was 19.9 ± 3.0 years among the dental students and 18.2 ± 1.2 years among the nursing students.

Table 1 shows the comparisons of attitudes towards oral healthcare among the dental and nursing students. After attending the courses, all dental students and almost all (98.0%) nursing students expressed interest in oral healthcare and a willingness to practise oral healthcare post-qualification. Approximately 60% of the dental students but only 24.3% of the nursing students expressed that the percentage of their duties that they expected to spend practising oral healthcare after post-qualification would be more than 50%. There were significant differences in interest in oral healthcare between both the dental students in their first and fourth years and the nursing students in their first and fourth years, and there were significant differences in the expected percentage of duties that would be constituted by oral healthcare practice both between the first- and fourth-year dental students and between the dental and nursing students.

**Table 1:** Comparisons of attitudes towards oral healthcare practice between students in their first and fourth years and between dental and nursing students

	Dental students (n = 48)			Nursing students (n = 103)			Dental versus nursing students	
	First year	Fourth year		First year	Fourth year		First year	Fourth year
	%	%	*P* value*	%	%	*P* value*	*P* value*	*P* value*
Are you interested in oral healthcare practice?								
Very much	31.3	41.7	0.010	19.4	38.8	0.003	0.557	0.255
Somewhat	37.5	52.1		44.7	45.6			
A little	25.0	6.3		27.2	13.6			
Not very much	6.3	0.0		7.8	1.9			
Not at all	0.0	0.0		1.0	0.0			
Do you think you want to practise oral healthcare after obtaining your professional post-qualification?								
Very much	29.2	29.2	0.364	5.8	28.2	< 0.000	< 0.000	0.545
Somewhat	47.9	54.2		36.9	50.5			
A little	16.7	16.7		39.8	19.4			
Not very much	6.3	0.0		16.5	1.9			
Not at all	0.0	0.0		1.0	0.0			
What proportion of your duties will be dedicated to practising oral healthcare post-qualification?								
≥ 75%	6.5	27.7	0.025	3.9	6.8	0.732	0.002	< 0.000
50–74%	43.5	31.9		18.4	17.5			
25–49%	37.0	21.3		38.8	34.0			
< 25%	13.0	19.1		38.8	41.7			

*Chi-squared test

Table 2 shows the comparisons of awareness of oral healthcare among dental and nursing students. More than 80% of the dental students perceived that they needed knowledge of general dentistry, general medicine, and nursing; in addition, awareness of the need for knowledge of general medicine in the dental students was significantly improved after they attended the courses (*P* < 0.01).

**Table 2:** Comparisons of awareness of oral healthcare between students in their first and fourth years and between dental and nursing students

	Dental students (n = 48)			Nursing students (n = 103)			Dental versus nursing students	
	First year	Fourth year	*P* value*	First year	Fourth year	*P* value*	First year	Fourth year
	%	%		%	%		*P* value*	*P* value*
What kinds of knowledge do you think are needed to practice oral healthcare?								
General dentistry	100.0	93.8	0.078	97.1	95.1	0.471	0.232	0.721
General medicine	58.3	83.3	0.007	44.7	72.8	< 0.000	0.118	0.158
Nursing	75.0	81.3	0.459	40.8	71.8	< 0.000	< 0.000	0.215
Geriatrics	41.7	58.3	0.102	47.6	90.3	< 0.000	0.498	< 0.000
Other	0.0	8.3	0.041	0.0	1.0	0.316	< 0.000	0.019
To whom do you think oral healthcare should be provided?								
Older adults who need nursing care	89.6	97.9	0.092	76.7	99.0	< 0.000	0.061	0.578
Healthy older adults	87.5	93.8	0.294	78.6	96.1	< 0.000	0.192	0.520
Patients in hospital wards	81.3	93.8	0.064	73.8	96.1	< 0.000	0.316	0.520
Healthy people, except for older adults	77.1	87.5	0.181	70.9	87.4	0.004	0.424	0.983
Cancer patients	66.7	83.3	0.059	39.8	96.1	< 0.000	0.002	0.007
Other	2.1	4.2	0.557	2.9	4.9	0.471	0.768	0.852
Where do you think oral healthcare should be provided?								
Long-term care facilities	79.2	83.3	0.601	79.6	95.1	0.001	0.950	0.016
Hospices	39.6	79.2	< 0.000	31.1	89.3	< 0.000	0.303	0.093
In the patient’s home	64.6	75.0	0.266	63.1	94.2	< 0.000	0.861	0.001
Recovery phase rehabilitation wards	52.1	66.7	0.146	34.0	91.3	< 0.000	0.034	< 0.000
Paediatric wards	56.3	66.7	0.294	58.3	92.2	< 0.000	0.817	< 0.000
Cancer wards	54.2	64.6	0.299	22.3	95.1	< 0.000	< 0.000	< 0.000
Acute care hospitals (including the ICU)	33.3	62.5	0.004	16.5	97.1	< 0.000	0.020	< 0.000
Maternity wards	20.8	58.3	< 0.000	16.5	93.2	< 0.000	0.518	< 0.000
Psychiatric wards	20.8	54.2	0.001	6.8	92.2	< 0.000	0.011	< 0.000
Other	0.0	2.1	0.315	2.9	1.9	0.651	0.232	0.954
What do you think oral healthcare affects?								
Prevention of periodontal disease	93.8	95.8	0.646	96.1	98.1	0.407	0.520	0.428
Prevention of aspiration pneumonia	58.3	95.8	< 0.000	35.9	97.1	< 0.000	0.010	0.688
Prevention of dental caries	93.6	93.8	0.979	98.1	99.0	0.561	0.160	0.060
Prevention of general disease	68.8	81.3	0.157	36.9	92.2	< 0.000	< 0.000	0.047
Care prevention (prevention of becoming frail)	47.9	79.2	0.001	32.0	86.4	< 0.000	0.060	0.257
Improvement of anorexia	52.1	75.0	0.020	31.1	93.2	< 0.000	0.013	0.002
Prevention of cardiovascular disease	66.7	70.8	0.660	44.7	84.5	< 0.000	0.012	0.051
Other	0.0	4.2	0.153	2.9	1.9	0.651	0.232	0.428

*Chi-squared test

More than 80% of the fourth-year dental and nursing students perceived that older adults and patients should be provided with oral healthcare. However, this perception was not significantly improved in the dental students after they attended the courses.

The only context where more than 80% of dental students perceived that oral healthcare should be provided was long-term facilities, while after they attended the courses, more than 80% of nursing students perceived that all contexts should provide oral healthcare. A significantly higher percentage of fourth-year nursing students than fourth-year dental students thought that all contexts except for hospices should provide oral healthcare (*P* < 0.05).

More than 90% of the dental and nursing students perceived the effectiveness of oral healthcare for preventing dental caries, periodontal diseases, and aspiration pneumonia, and awareness of the effectiveness of oral healthcare for the prevention of aspiration pneumonia, care prevention (prevention of becoming frail), and anorexia was significantly improved in both the nursing and dental students after they attended the courses (*P* < 0.05).

Tables 3 and 4 show the comparisons of the dental and nursing students’ perceptions of the aspects of oral healthcare practice that they needed to learn in theory and in practice. Only the perception of the need to have language training in both theory and practice was significantly improved in the dental students (*P* < 0.01), while the perceptions of the need to learn many types of procedures in both theory and practice were significantly improved in the nursing students after they attended the courses (*P* < 0.05).

**Table 3:** Comparisons of perceptions of aspects of oral healthcare practice that need to be learned (in theory) between students in their first and fourth years and between dental and nursing students

	Dental students (n = 48)			Nursing students (n = 103)			Dental versus nursing students	
	First year	Fourth year		First year	Fourth year		First year	Fourth year
	%	%	*P* value*	%	%	*P* value*	*P *value*	*P* value*
What kinds of treatments or instructions of oral healthcare do you think you need to learn in lecture?								
Support of tooth brushing	75.0	87.5	0.117	67.0	89.3	< 0.000	0.319	0.742
Use of an interspace brush	85.4	83.3	0.779	78.6	76.7	0.738	0.325	0.353
Swabbing of oral soft issue	75.0	75.0	1.000	72.8	82.5	0.094	0.777	0.281
Removal tongue coating	58.3	75.0	0.083	47.6	70.9	0.001	0.218	0.598
Indirect training for swallowing	58.3	75.0	0.083	20.4	77.7	< 0.000	< 0.000	0.717
Direct training for swallowing (using foods and drinks)	58.3	72.9	0.133	21.4	72.8	< 0.000	< 0.000	0.990
Gargling	60.4	70.8	0.283	49.5	66.0	0.016	0.211	0.556
Denture cleaning	62.5	70.8	0.386	34.0	71.8	< 0.000	0.001	0.898
Salivary gland massage	54.2	66.7	0.210	34.0	86.4	< 0.000	0.019	0.005
Oral management in the perioperative ward	52.1	64.6	0.214	31.1	84.5	< 0.000	0.013	0.006
Language training	31.3	62.5	0.002	18.4	55.3	< 0.000	0.079	0.407
At-home dental care	52.1	62.5	0.302	50.5	73.8	0.001	0.855	0.158
Other	2.1	8.3	0.168	1.9	1.9	1.000	0.954	0.061

*Chi-squared test

**Table 4:** Comparisons of perceptions of aspects of oral healthcare practice that need to be learned (in practice) between students in their first and fourth years and between dental and nursing students

	Dental students (n = 48)			Nursing students (n = 103)			Dental versus nursing students	
	First year	Fourth year		First year	Fourth year		First year	Fourth year
	%	%	*P* value*	%	%	*P* value*	*P* value*	*P* value*
What kinds of treatments or instructions of oral healthcare do you think you need to learn in practice?								
Support of tooth brushing	70.8	87.5	0.044	76.7	95.1	< 0.000	0.439	0.092
Use of an interspace brush	85.4	81.3	0.584	80.6	79.6	0.861	0.470	0.814
Indirect training for swallowing	58.3	75.0	0.083	29.1	70.9	< 0.000	0.001	0.598
Direct training for swallowing (using foods and drinks)	56.3	75.0	0.053	26.2	68.9	< 0.000	< 0.000	0.445
Swabbing of oral soft issue	72.9	72.9	1.000	72.8	82.5	0.094	0.990	0.174
Removal of tongue coating	66.7	72.9	0.505	50.5	73.8	0.001	0.062	0.910
Gargling	60.4	72.9	0.194	53.4	66.0	0.065	0.419	0.397
Salivary gland massage	58.3	70.8	0.200	39.8	87.4	< 0.000	0.033	0.013
Language training	37.5	66.7	0.004	18.4	65.0	< 0.000	0.011	0.846
Oral management in the perioperative ward	47.9	64.6	0.100	33.0	73.8	< 0.000	0.078	0.247
Denture cleaning	62.5	62.5	1.000	41.7	74.8	< 0.000	0.017	0.123
Domiciliary dental care	52.1	60.4	0.411	46.6	57.3	0.125	0.530	0.716
Other	2.1	10.4	0.092	1.9	1.9	1.000	0.954	0.021

*Chi-squared test

Table 5 shows the comparison of the levels of attitudes, awareness, and perceptions regarding oral healthcare among the dental and nursing students. The levels of interest in and awareness of oral healthcare were significantly higher in both the fourth-year dental and fourth-year nursing students than in the first-year dental and first-year nursing students (*P* < 0.01), while the levels of perception of the need to learn procedures in oral healthcare were significantly improved in only the nursing students (*P* < 0.001). The levels of interest in and awareness of oral healthcare and the perception of the importance of collaboration with healthcare workers such as physicians, nurses, and speech-language-hearing therapists were higher in the fourth-year nursing students than in the fourth-year dental students.

**Table 5:** Comparisons of levels of attitudes, awareness, and perceptions regarding oral healthcare between students in their first and fourth years and between dental and nursing students

	Dental students (n = 48)			Nursing students (n = 103)			Dental versus nursing students	
	First year	Fourth year		First year	Fourth year		First year	Fourth year
	Mean (SD)	Mean (SD)	*P* value*	Mean (SD)	Mean (SD)	*P* value*	*P* value**	*P* value**
Level of interest in oral healthcare practice (0–4 point)								
	2.9 (0.9)	3.4 (0.6)	0.002	2.7 (0.9)	3.2 (0.7)	< 0.000	0.214	0.374
Level of willingness to practise oral healthcare after professional certification (0–4 point)								
	3.0 (0.9)	3.1 (0.7)	0.283	2.3 (0.9)	3.0 (0.7)	< 0.000	< 0.000	0.627
Level of awareness of oral healthcare (0–29 point)								
	15.8 (5.6)	19.9 (5.8)	< 0.000	12.8 (4.5)	23.0 (4.1)	< 0.000	0.001	< 0.000
Levels of perception of need to learn different procedures in oral healthcare								
In theory (0–14 point)	7.3 (4.2)	8.8 (4.3)	0.056	5.3 (3.3)	9.1 (3.4)	< 0.000	0.006	0.881
In practice, (0–14 point)	7.3 (4.3)	8.7 (4.4)	0.056	5.7 (3.3)	9.0 (3.5)	< 0.000	0.019	0.693
Total (0–28 point)	14.6 (8.4)	17.5 (8.4)	0.054	11.0 (6.3)	18.1 (6.5)	< 0.000	0.010	0.699
Levels of perception of the importance of practising collaborative oral healthcare with healthcare workers***								
Physicians (0–3 point)	–	2.3 (0.7)	–	–	2.6 (0.6)	–	–	0.007
Dentists (0–3 point)	–	2.9 (0.4)	–	–	3.0 (0.1)	–	–	0.060
Dental hygienists (0–3 point)	–	2.9 (0.4)	–	–	3.0 (0.2)	–	–	0.137
Nurses (0–3 point)	–	2.5 (0.5)	–	–	2.9 (0.4)	–	–	< 0.000
Certified care workers (0–3 point)	–	2.7 (0.5)	–	–	2.6 (0.5)	–	–	0.353
Speech–language–hearing therapists (0–3 point)	–	2.4 (0.7)	–	–	2.8 (0.5)	–	–	< 0.000
Total (0–18 point)	–	15.7 (2.4)	–	–	16.8 (1.5)	–	–	0.006

*Wilcoxon signed-rank test

**Mann–Whitney U test

***These questions were asked only to fourth-year students

## Discussion

This follow-up and cross-sectional study was the first to compare attitudes, awareness, and perceptions regarding oral healthcare among dental and nursing students after oral healthcare education. The results showed that interest in practising and willingness to practise oral healthcare in both the dental and nursing students were significantly improved after the oral healthcare courses. This study confirmed the results of previous studies that showed that multi-professional education for healthcare professional students was effective in improving attitudes. In addition, the dental students seemed to perceive that oral healthcare practice was one of the main duties of practice better than the nursing students. A previous study reported that interest in oral healthcare was significantly associated with the willingness to practise it post-qualification [18]. Therefore, the improvement in interest might have affected their willingness to practise oral healthcare as a main duty.

After the oral health education, more than 80% of the dental students perceived that they needed to learn general dentistry, general medicine, and nursing to practise oral healthcare, more than 90% perceived that oral healthcare should be provided to older adults, more than 80% perceived that long-term care facilities should be provided, and more than 95% perceived the preventive effectiveness of aspiration pneumonia. These findings showed that they might have perceived that oral healthcare practice for older adults would be one of their main practices post-qualification, as Japanese society is ageing [29].

However, the awareness of the need to provide oral healthcare to cancer patients and the awareness of contexts where oral healthcare should be provided were lower in the dental students than the nursing students. In addition, the level of awareness regarding oral healthcare was also lower in the dental students. Case-based learning of oral healthcare practice for patients in paediatric, cancer, perioperative, convalescent, psychiatric, and palliative care wards in hospitals was introduced in nursing oral healthcare courses. The learning might be effective in improving awareness [26, 30]. In addition, the nursing students had participated in clinical training at various wards in some hospitals from the first to fourth years during the study period, and they might have observed nurses’ oral healthcare practice there. Therefore, it is suggested that case-based learning, including oral healthcare practice, for patients in various hospital wards should be introduced in dental oral healthcare education, but further studies are needed to prove the effectiveness of such education for dental students.

The dental students’ perceptions of the need for learning most procedures and patient instructions in oral healthcare were not significantly improved after the courses. They might have believed that they could learn these procedures and instruction in the existing dental courses without a special oral healthcare programme. However, the main purpose of oral healthcare practice differs across cases. For example, different main purposes have been reported, including the prevention of VAP in patients in intensive care units (ICUs) and perioperative wards [1], the relief of oral pain in patients treated with radiotherapy [31], and the prevention of aspiration pneumonia in patients with dysphagia [2]. Therefore, it is suggested that oral healthcare education should be designed to help dental students deal with a variety of cases in oral healthcare practice post-qualification.

The levels of perception of the importance of collaboration with physicians, nurses, and speech–language–hearing therapists in oral healthcare were lower in the dental students than in the nursing students. Physicians and nurses play the main roles in providing healthcare, including oral healthcare for patients in hospitals, older adult patients in long-term care facilities, and community-dwelling older adult patients [8–15, 32]. Furthermore, nurses can also encourage patients to undergo dental treatments and receive care from oral health professionals [33, 34]. Therefore, it is suggested that dental students should perceive the importance of collaborating with health professionals in oral healthcare practice post-qualification. A previous study reported that multi-professional education with dental and nursing students was effective in improving their interest in oral health [21, 22]; therefore, the education may also be effective in improving perception. In addition, it is suggested that nurses should be included in multi-professional oral healthcare education for dental students to improve their perception.

There are several limitations associated with this study. First, one Japanese dental school and one Japanese nursing school were investigated, and the sample of students was selected without a power calculation in this study. There were 29 dental schools and 267 nursing schools in Japan at the time of the study [35]. The 45-h oral healthcare programmes taught by multiple professionals in the nursing school were somewhat unique compared to the programmes offered by other nursing schools, as only 5.1% of nursing schools were reported to have a special course for oral healthcare [36]. Therefore, the results of this study are not generalizable, and if other students from other nursing schools had been recruited in this study, the results might have been completely different. However, recruiting nursing students who participated in special oral healthcare courses was better than recruiting nursing students from other nursing schools to explore the problems of oral healthcare education in dental curricula. Second, the medical and other dental courses in the dental school and the medical and nursing courses in the nursing school that the participants took might have affected them during the study period. Nursing clinical training in hospitals might have affected the differences in awareness and perceptions regarding oral healthcare practice between the dental and nursing students. Third, the response rates were 58.3% in the dental students and 91.2% in the nursing students, and this difference might have affected the results in this study because people interested in the topic were more likely to respond than those who were not interested [37]. Fourth, perceptions of collaboration with other healthcare professionals might have been higher in the nursing students than the dental students regardless of their education because nurses need to provide care for their patients collaborating with other health professionals [26, 38].

## Conclusion

In conclusion, the levels of awareness regarding oral healthcare and the perceptions of the importance of collaboration among health professionals were higher in the nursing students, who had a special oral healthcare course that was composed of case-based learning programmes taught by multiple professionals, than in the dental students, who had no case-based learning programme. Therefore, unified oral healthcare programmes with case-based learning taught by multiple professionals from different disciplines might also be effective in improving awareness and perceptions regarding oral healthcare practice among dental students, but further studies are needed to prove the effectiveness of such programmes.

The results of this study showed that almost all dental and nursing students were interested in oral healthcare practice and perceived the preventive effect of oral healthcare on aspiration pneumonia, and their interest and awareness were significantly improved after they attended the oral healthcare courses. However,

## Supplementary Information

**Additional file 1.** STROBE Statement—Checklist of items that should be included in reports of cross-sectional studies.

## Data Availability

The datasets generated and/or analysed during the current study are not publicly available as ethics approval was granted on the basis that only the researchers involved in the study could access the identified data but are available from the corresponding author on reasonable request.

## Abbreviations

VAP: Ventilator-associated pneumonia
ICU: Integrated circuit unit
